# Subject-Specific Spino-Pelvic Models Reliably Measure Spinal Kinematics During Seated Forward Bending in Adult Spinal Deformity

**DOI:** 10.3389/fbioe.2021.720060

**Published:** 2021-09-01

**Authors:** Thomas Overbergh, Pieter Severijns, Erica Beaucage-Gauvreau, Thijs Ackermans, Lieven Moke, Ilse Jonkers, Lennart Scheys

**Affiliations:** ^1^ Department of Development and Regeneration, Faculty of Medicine, Institute for Orthopaedic Research and Training (IORT), KU Leuven, Leuven, Belgium; ^2^ Division of Orthopaedics, University Hospitals Leuven, Leuven, Belgium; ^3^ Department of Movement Sciences, Human Movement Biomechanics Research Group, KU Leuven, Leuven, Belgium

**Keywords:** spine kinematics, reliability, operator variability, adult spinal deformity, motion analysis, opensim model, subject-specific modeling and simulation, spine model

## Abstract

Image-based subject-specific models and simulations are recently being introduced to complement current state-of-the-art mostly static insights of the adult spinal deformity (ASD) pathology and improve the often poor surgical outcomes. Although the accuracy of a recently developed subject-specific modeling and simulation framework has already been quantified, its reliability to perform marker-driven kinematic analyses has not yet been investigated. The aim of this work was to evaluate the reliability of this subject-specific framework to measure spine kinematics in ASD patients, in terms of 1) the overall test-retest repeatability; 2) the inter-operator agreement of spine kinematic estimates; and, 3) the uncertainty of those spine kinematics to operator-dependent parameters of the framework. To evaluate the overall repeatability 1], four ASD subjects and one control subject participated in a test-retest study with a 2-week interval. At both time instances, subject-specific spino-pelvic models were created by one operator to simulate a recorded forward trunk flexion motion. Next, to evaluate inter-operator agreement 2], three trained operators each created a model for three ASD subjects to simulate the same forward trunk flexion motion. Intraclass correlation coefficients (ICC’s) of the range of motion (ROM) of conventional spino-pelvic parameters [lumbar lordosis (LL), sagittal vertical axis (SVA), thoracic kyphosis (TK), pelvic tilt (PT), T1-and T9-spino-pelvic inclination (T1/T9-SPI)] were used to evaluate kinematic reliability 1] and inter-operator agreement 2]. Lastly, a Monte-Carlo probabilistic simulation was used to evaluate the uncertainty of the intervertebral joint kinematics to operator variability in the framework, for three ASD subjects 3]. LL, SVA, and T1/T9-SPI had an excellent test-retest reliability for the ROM, while TK and PT did not. Inter-operator agreement was excellent, with ICC values higher than test-retest reliability. These results indicate that operator-induced uncertainty has a limited impact on kinematic simulations of spine flexion, while test-retest reliability has a much higher variability. The definition of the intervertebral joints in the framework was identified as the most sensitive operator-dependent parameter. Nevertheless, intervertebral joint estimations had small mean 90% confidence intervals (1.04°–1.75°). This work will contribute to understanding the limitations of kinematic simulations in ASD patients, thus leading to a better evaluation of future hypotheses.

## Introduction

Musculoskeletal (MS) models and associated simulations of motion are used to provide a better understanding of the complex biomechanics of, primarily, the healthy spine (Bruno et al., 2015; Ignasiak et al., 2018; Beaucage-Gauvreau et al., 2019). These simulation-based approaches provide parameters that are otherwise difficult, or even impossible, to measure non-invasively *in vivo*, such as intervertebral (IV) joint angles, IV disc loads (Bruno et al., 2017) and spinal muscle forces (Burkhart et al., 2017). Indeed, in healthy subjects these MS models have shown excellent test-retest reliability in terms of spine curvature estimation (expressed as lumbar lordosis and thoracic kyphosis) (Burkhart et al., 2020). More recently, these MS models and simulation-based approaches were introduced in pathological spine populations, such as adult spinal deformity (ASD) (Overbergh et al., 2020) and adolescent idiopathic scoliosis (AIS) (Schmid et al., 2016), to complement the current state-of-the-art mostly static assessments and on the longer term improve the often poor outcomes of surgical treatments (Smith et al., 2016). More specifically, a novel method based on biplanar radiography and computed tomography (CT) was developed to create subject-specific spino-pelvic rigid body models that allows inclusion of personalized spinal alignment, intervertebral joint definitions, and associated virtual skin markers for ASD patients (Overbergh et al., 2020). The resulting subject-specific models from this method can provide innovative, functional biomarkers of pathological spine biomechanics. This novel modeling method circumvents the traditional marker-based scaling step (Delp et al., 2007; Burkhart et al., 2020), which is applicable to healthy subjects, but not suitable for subjects with a spinal malalignment due to the lack of sufficient a priori information on the specific spinal deformity.

However, to improve the rigor and objectivity of the results prior to clinical interpretation, it is imperative to verify the simulation results of modeling methods both in terms of accuracy and reliability (Schwartz et al., 2004; Hicks et al., 2015). The accuracy of the above-mentioned subject-specific biplanar radiograph-based modeling method, as well as its accuracy in estimating spine kinematics, was validated previously (Overbergh et al., 2020).

Nevertheless, the subject-specific model creation method and the use of these subject-specific models to evaluate spinal kinematics remain susceptible to variability from different sources of errors and the impact thereof has not been investigated yet. Indeed, the creation of image-based subject-specific spino-pelvic models requires operator-dependent manual inputs to define virtual markers, spinal alignment, and IV joints (Overbergh et al., 2020), resulting in an extrinsic variability on the simulation outputs (Schwartz et al., 2004). The reliability of these operator-dependent inputs can be evaluated using an operator agreement analysis quantifying the robustness of the kinematic simulation results to this extrinsic variability (Hicks et al., 2015). In addition, the reliability of the kinematics of a subject is affected by intra-subject differences (i.e., within- or between-session variability), categorized as intrinsic variability (Schwartz et al., 2004). In relation to this intrinsic variability, the test-retest reliability of spino-pelvic parameterization through marker-based polynomial fitting of a sit-to-stance (STS) motion has already been investigated in an ASD population, and was reported to perform equally or even more reliable than conventional radiographic measurements (Severijns et al., 2020). However, the effect of these intra-subject differences in combination with image-based subject-specific models has not yet been investigated in an ASD population.

Specifically for biomechanical modeling and simulation research, the complex non-linear interactions between input and output parameters often require an extension to the conventional operator agreement analyses to obtain a representative range of output variability and identify the aspects of the modeling method that have the highest/lowest impact on the outputs (Hicks et al., 2015). Therefore, uncertainty analyses, such as Monte-Carlo probabilistic simulations, are commonly used to assess the simultaneous impact of uncertainties arising from multiple sources (Hicks et al., 2015; Myers et al., 2015). Monte-Carlo analyses allow computation of sensitivity factors (e.g., correlation coefficients) to determine relations between the input and output distributions (Hicks et al., 2015; Myers et al., 2015) to identify the modeling components with a high impact on the output for future improvements. Thereto, Monte Carlo analyses generate a large number of statistically probable variations of a baseline model, consisting of randomly combined perturbations of the operator-dependent parameters susceptible to uncertainty. These perturbations are sampled from a probability density function representative of the actual variability of the operator-dependent parameters (Valero-Cuevas et al., 2003; Hannah et al., 2017). The impact of these operator-dependent parameters on the simulation outputs can then be translated into confidence bounds on the baseline output (Ackland et al., 2012; Valente et al., 2014; Myers et al., 2015).

The aim of this study was to evaluate the reliability of a previously developed subject-specific spino-pelvic modeling method (Overbergh et al., 2020) to measure spine kinematics in an ASD population, in terms of 1) the overall test-retest repeatability and 2) the inter-operator agreement of spine kinematic estimates; and 3) the sensitivity of those spine kinematics to operator-dependent aspects of the underlying subject-specific modeling method.

## Materials and Methods

### Participants and Data Collection

Five participants [2 males (51 and 72 years), 3 females (62, 69, and 70 years)] with varying degrees of spinal malalignment and one control subject (female) participated in this study following ethical approval and informed consent (S58082) (Overbergh et al., 2020). All data collection was performed in at the university hospital of Leuven (UZ Leuven, Belgium). All subjects underwent CT imaging from T1 to pelvis (BrightSpeed by GE Healthcare, with an inter-slice distance of 1.25 mm and a pixel size of 0.39 mm × 0.39 mm). Thereafter, an experienced physiotherapist instrumented each subject with reflective markers according to the skin marker protocol described in Overbergh et al. (2020). Full-body radiographic (x-ray) images were then acquired using the biplanar radiography system (EOS Imaging, Paris, France), while the subject was wearing the markers and adopted the Scoliosis Research Society free-standing position (fingers-on-clavicle variation) (Wang et al., 2014). When the subjects arrived at the motion laboratory, they were asked to perform a maximal forward trunk flexion from a normal upright seated position, while the trajectories of the reflective markers were recorded (100 Hz) using a 10-camera Vicon system (VICON Motion systems, Oxford Metrics, United Kingdom). Four of the five ASD patients and the control subject repeated all data collections, apart from the CT imaging, after an average 2-week time interval (mean 14.2 ± 9.9 days, 6–33 days). One ASD patient (male) was excluded for the second data collection due to a surgical intervention, but remained part of the study because of a successful first data collection.

### Test-Retest Reliability

To test the repeatability of our workflow for spinal kinematic evaluation, we performed a test-retest reliability analysis between the two repeated data collection sessions available for each of the four ASD subjects (one excluded) and the control subject. Two subject-specific spino-pelvic models were created by one single operator to prevent confounding inter-rater variability; one for the initial data collection and one for the repeated data collection, respectively (Overbergh et al., 2020). The resulting subject-specific spine models each consist of 18 bodies (12 thoracic vertebrae, 5 lumbar vertebrae and a sacrum/pelvis body), interconnected by 17 spherical joints [each with three rotational degrees of freedom (DOFs)] and have a total of 28 virtual model markers each, corresponding to the retroreflective markers placed on the skin of the subject (Overbergh et al., 2020). It should be noted that these aspects of the model (i.e., bodies, joints and markers) all required input from an operator (Overbergh et al., 2020). The maximal forward trunk flexion motion, recorded as three-dimensional (3D) marker trajectories in the motion laboratory, was processed using Vicon Nexus 2.11 (VICON Motion systems, Oxford Metrics, United Kingdom) and low-pass Butterworth filtered (6 Hz). For each subject and each session, the respective models were used to run an inverse kinematics analysis (Lu and O’Connor, 1999) in OpenSim 3.3 (Stanford University, United States) (Delp et al., 2007) of the corresponding forward trunk flexion motions. The kinematic outputs (i.e., 51 joint angles ranging from L5/Sacrum to T1/T2) were time-normalized (to 100 frames) and noise reduction was performed using a moving average filter with a three-frame width. The joint kinematics (i.e., relative motion at the joint between two interconnected bodies) were converted to body kinematics (i.e., absolute motion of a body expressed in the ground reference frame) to obtain six common spino-pelvic parameters in the sagittal plane based on a-priori identification of anatomical landmarks on the model: 1) lumbar lordosis (LL), 2) thoracic kyphosis (TK), 3) sagittal vertical axis (SVA), 4) pelvic tilt (PT), and 5) T1 and 6) T9 spino-pelvic inclination (T1-SPI, T9-SPI), (detailed in Supplementary Appendix S1). The ranges of motion (ROM) of each of these spino-pelvic parameters (defined as the absolute value of the difference between the start and the end of the motion, Supplementary Appendix S1) were used as an outcome parameter to determine the test-retest reliability. This test-retest reliability was expressed as intraclass correlation coefficients (ICC’s) with a two-way random effects model for absolute agreement [ICC(2,1)] (SPSS 25, IBM Corp. Armonk, NY). ICC’s were classified as poor (ICC <0.40), fair to good (0.40–0.75) or excellent (>0.75) (Shrout and Fleiss, 1979). Standard error of measurement (SEM) was calculated as:

$$SEM=SD\;\sqrt{1-ICC},$$ (1)

with SD the standard deviation of the absolute difference relative to the mean output; and the smallest detectable difference (SDD) (Shrout and Fleiss, 1979) as:

$$SDD=SEM\times 1.96\;\sqrt{2}$$ (2)

### Inter-Operator Reliability

**FIGURE 1:**
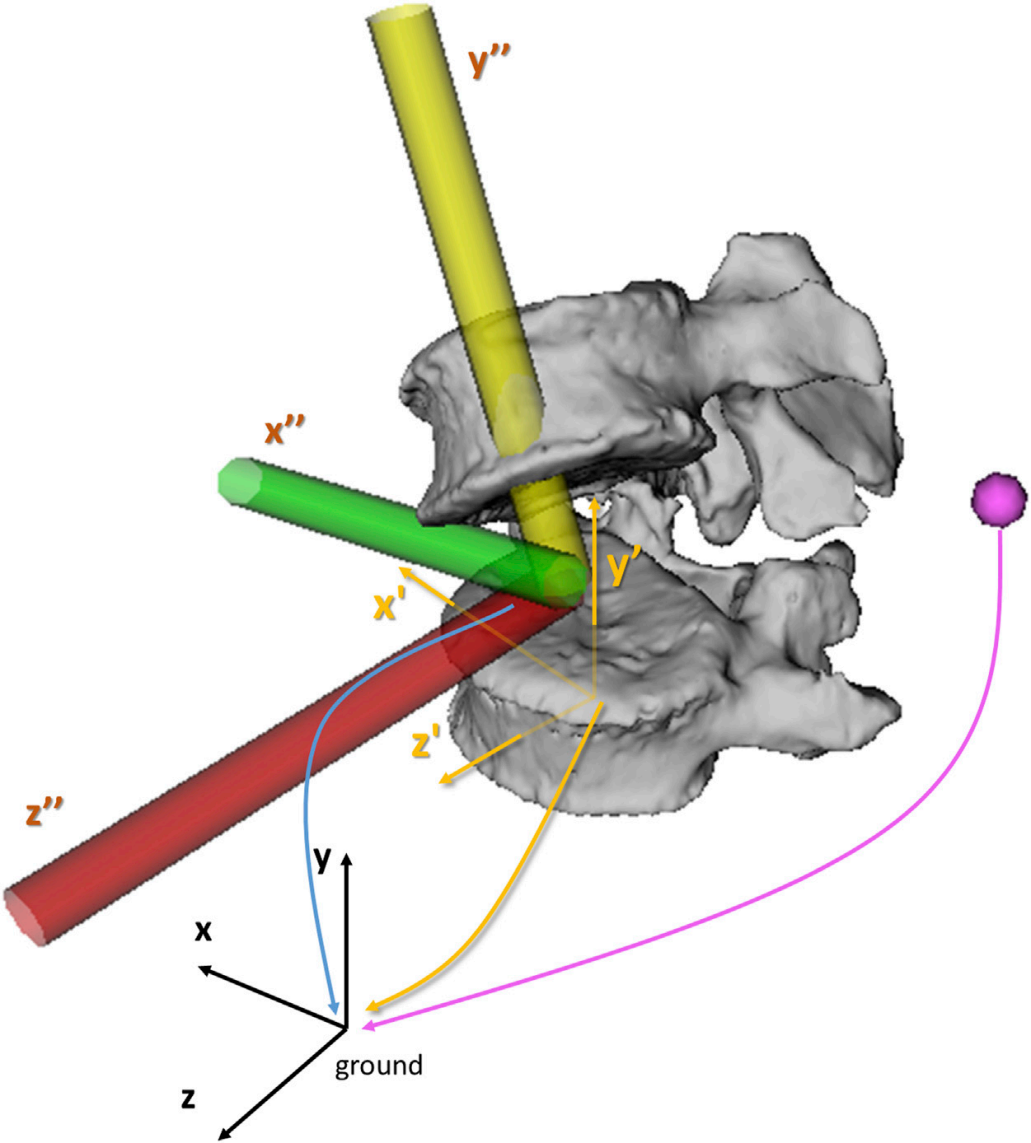
Illustration of the three operator-dependent parameters components. The position of the virtual markers (pink sphere), the position and orientation of the bodies (yellow reference frame, x’y’z’) and the position and orientation of the IV joints (yellow, green and red reference frame, x”y”z”) are expressed in the ground reference frame (black, xyz). Within the model, positions of virtual markers, bodies and joints, are expressed in the x (mediolateral), y (inferosuperior) and z (posterior-anterior) directions. The orientations of the joints and bodies are expressed around the x (flexion-extension, FE), y (axial rotation, AR) and z-axis (lateroflexion, LF) using an xyz body-fixed sequence.

To assess the portion of variability of the modeling method on the kinematic results that can be attributed to operator-dependent inputs (Overbergh et al., 2020), three operator-dependent modeling components (and their associated parameters) were first identified (Figure 1): (A) virtual markers (position parameters): the reconstruction of virtual marker positions requires operators to identify and delineate retro-reflective markers on both biplanar radiographic images; (B) bodies (i.e., vertebrae and pelvis) (position and orientation parameters): the manual reconstruction of the 3D spinal alignment requires operators to match subject-specific vertebrae projections on biplanar radiographic images until visual agreement; (C) joints (position and orientation parameters): the IV joint definition requires operators to manually identify anatomical landmarks on the bodies connected by these joints. This results in a total of 294 operator-dependent parameters [(28 markers × 3 DOFs) + (18 bodies × 6 DOFs) + (17 joints × 6 DOFs)].

**FIGURE 2:**
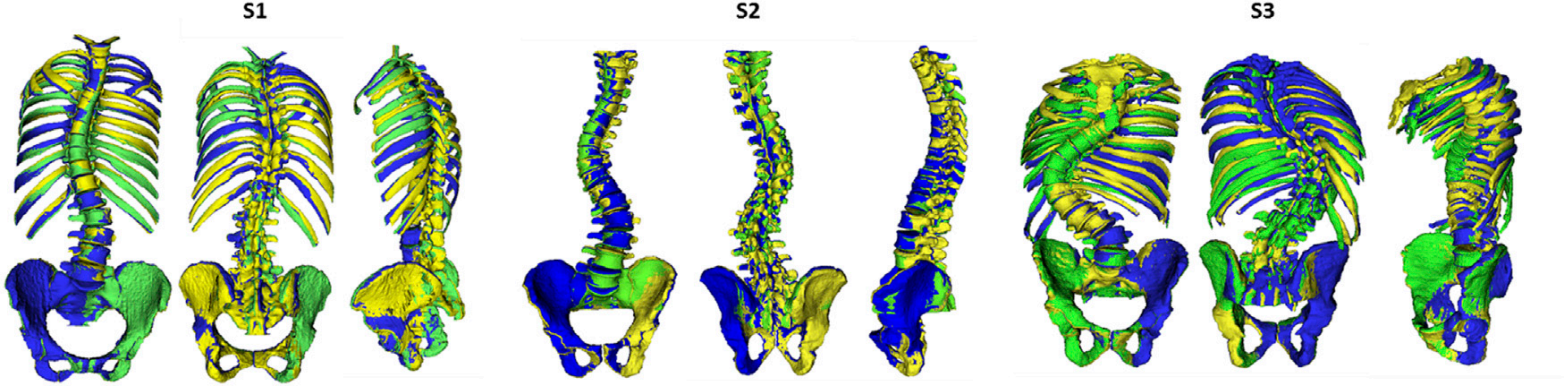
Illustration of the alignment reconstruction for the three subjects [S1 (female), S2 (male) and S3 (male)] by the three operators: O1 (green), O2 (yellow) and O3 (blue).

Three operators participated in this study. One operator (O1, 4 years of spine modeling experience and developer of the modeling method), trained two additional operators (O2 and O3 with 6 and 2 years of spinal research experience, respectively) on the required steps of the modeling workflow through a dedicated manual describing optimal use of the custom software. Next, radiographic data of a cadaver with known ground truth spinal alignment due to plastination, was used for acquainting with and training in spinal alignment personalization (Overbergh et al., 2020) (detailed in Supplementary Appendix S2), followed by a final collective, quantitative feedback session between the operators. Then, each operator created a subject-specific spinal model of three randomly selected subjects (S1, S2 and S3, Figure 2) from the ASD group while being blinded to the other operators. The models were created as described in the modeling workflow of Overbergh et al. (2020), with the exception of segmenting the individual bones from CT which was only performed only by O1.

#### Inter-Operator Agreement

Each of the nine created models was used to perform an inverse kinematics simulation of the subject’s corresponding maximal forward flexion motion to obtain the ROM values for the six spino-pelvic parameters (LL, TK, SVA, PT, T1-SPI, T9-SPI). ICC’s, SEM (Formula 1) and SDD (Formula 2) on these outcome values were used to assess inter-operator agreement.

#### Monte-Carlo Probabilistic Simulation

**FIGURE 3:**
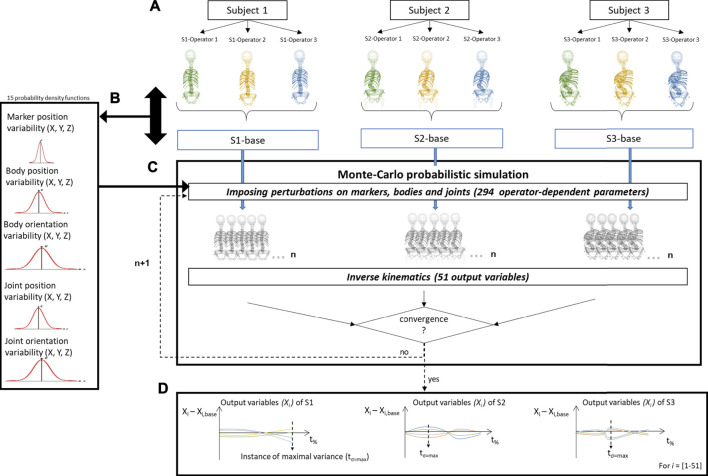
Schematic representation of the determination of the inter-operator reliability of subject-specific modeling. **(A)** For each subject (S), a subject-specific model was created by each of the three operators (O). A baseline model (S-base) was then created for every subject by averaging these three respective models. **(B)** The variability in the operator-dependent parameters was calculated in relation to the respective baseline models, pooled together for all vertebral levels and subjects, and separated by direction. **(C)** In the Monte-Carlo probabilistic simulation, variations on the baseline model were created by imposing statistically probable error combinations on the operator-dependent parameters and then used to perform inverse kinematic simulations until the convergence criterion on the output variables (i.e., the joint angles) was reached. **(D)** The joint angles (X_i_ with i = 1...51) were then expressed relative to the joint angles of the corresponding baseline model (X_i, base_) and time normalized (t_%_). t_σ=max_ represents the time instance of maximal variance.

We performed a Monte-Carlo probabilistic simulation analysis to quantify the distributions of variations on simulated IV joint kinematics caused by operator variability, similar to the work by Valente et al. (2014). First, a baseline model (S-base) was determined for each of the three ASD subjects to avoid operator bias, by averaging the three operator-defined models (Figure 3A). These baseline models were considered as reference models to experimentally estimate the variability of the 294 operator-dependent model parameters. The variations of these operator-dependent components (marker, bodies, and joint) for the three models with respect to its respective baseline model were pooled into histograms over all vertebral levels and subjects, and separated by direction (x, y, z) for each parameter (position and orientation) (Supplementary Appendix S3). From these experimentally determined variability histograms, continuous probability density functions were estimated (MATLAB, The Mathworks Inc., MA) (Supplementary Appendix S3), and used as input to sample variations on the 294 operator-dependent model parameters (Figure 3B). This ensured statistically probable imposed perturbations according to a-priori experimentally determined inter-operator variability. To create a perturbed model, a value was sampled from the probability function for each operator-dependent model parameter and used to vary the value of that parameter in the baseline model. For each subject, every variation of the baseline model was then used to run an inverse kinematics analysis (Lu and O’Connor, 1999) (Figure 3C). The convergence criterion for the Monte-Carlo simulation was defined such that the mean and standard deviation of all output variables (here: joint angles averaged over the duration of the motion) over the last 10% of the simulations were within 2% of each final mean and standard deviation (Supplementary Appendix S4) (Valero-Cuevas et al., 2003; Ackland et al., 2012; Valente et al., 2013; Martelli et al., 2015).

##### Operator-Dependent Input Parameters

After assessing normality of the parameters of the model components (position and/or orientation of markers, bodies, joints), kernel functions were consistently used to estimate all distribution functions from their respective histograms (Distribution Fitter, MATLAB, The Mathworks Inc., MA) (Supplementary Appendix S3). To assess the variation of the operator-dependent inputs in the modeling method (markers, bodies and joints), we used the absolute value of the difference between each of the three operator-dependent models and its baseline model to determine the median and maximum values for each individual position and orientation parameter, in each direction.

To assess the robustness of the IV joint kinematics to variations in the operator-dependent model parameters, joint angles of the perturbed models were expressed relative to the joint angles of the baseline model’s kinematics. For each subject, we then determined the 5–95% confidence bounds for each of the joint angles (17 joints with three rotational DOFs each), at each time frame of the performed spine flexion motion, which indicates a 90% probability that an estimated joint angle curve is within the confidence intervals with respect to the calculated reference curve (Myers et al., 2015; Navacchia et al., 2016). Thereafter, a box and whiskers plot was created at the time instance of respective maximal variance (t_σ=max,_ Figure 3D) for every DOF at every joint (Ackland et al., 2012).

##### Sensitivity Factors

To quantify the sensitivity of simulated kinematics to variability in specific input parameters, sensitivity factors were determined as Pearson correlation coefficients (Myers et al., 2015) between the sampled perturbation values (for each of the 294 model parameter) and the corresponding absolute maximal difference of the IV joint kinematics with respect to the baseline model’s IV joint kinematics (for each of the 51 DOFs), pooled for all three subjects (MATLAB).

## Results

### Test-Retest Reliability

The test-retest reliability, expressed as ICCs of six spino-pelvic parameters in Table 1, was excellent (ICC>0.75) for the LL, SVA, PT (not significant), T1-SPI and T9-SPI. Nevertheless, high SEM and SDD were noted for TK, which presented with a poor reliability (ICC<0.40).

**TABLE 1:** Results of the test-retest reliability analysis. ROM, range of motion; ICC, intraclass correlation coefficient; SD, standard deviation of the absolute differences between both sessions; SEM, standard error of measurement; SDD, smallest detectable difference. Significance level: *p* < 0.05 (bold). The confidence intervals for ICC’s with a non-significant *p* value are not applicable.

Spino-pelvic parameter ROM	Test-retest ICC	95% confidence interval	*p* value	SD	SEM	SDD	Mean (range) ROM
LL (°)	0.86	0.032–0.985	**0.028**	5.5	2.1	5.7	20.5 (9.5–42.4)
TK (°)	0.12	—	0.460	6.2	5.8	16.1	19.8 (1.8–30.9)
SVA (cm)	0.91	0.363–0.991	**0.018**	0.9	0.3	0.7	30.0 (25.4–40.6)
PT (°)	0.80	—	0.095	5.3	2.4	6.6	53.9 (30.4–60.4)
T1-SPI (°)	0.91	0.226–0.990	**0.012**	4.7	1.4	4.0	66.7 (46.1–89.7)
T9-SPI (°)	0.91	0.360–0.990	**0.015**	4.7	1.4	3.9	60.6 (39.5–81.7)

### Inter-Operator Agreement

Excellent inter-operator agreement (ICCs ≥0.875) of the kinematics, expressed as spino-pelvic parameters, was noted for all analyzed parameters (Table 2).

**TABLE 2:** Results of the inter-operator reliability analysis. ROM, range of motion; ICC, intraclass correlation coefficient, SD, standard deviation of absolute error relative to mean value; SEM, standard error of measurement; SDD, smallest detectable difference. Significance level: *p* < 0.05 (bold).

Spino-pelvic parameter ROM	Inter-operator ICC	95% confidence interval	*p* value	Mean SD	SEM	SDD
LL (°)	0.970	0.775–0.999	0.002	1.82	0.3	0.9
TK (°)	0.875	0.189–0.997	0.031	1.95	0.7	1.9
SVA (cm)	0.964	0.737–0.999	0.005	0.43	0.1	0.2
PT (°)	0.998	0.981–1.000	<0.001	0.13	0.0	0.0
T1-SPI (°)	1.000	0.999–1.000	<0.001	0.06	0.0	0.0
T9-SPI (°)	1.000	0.998–1.000	<0.001	0.07	0.0	0.0

### Monte-Carlo Probabilistic Simulation

#### Operator-dependent Input Parameters

The median difference in the virtual marker positions with respect to the baseline models ranged between 0.120 and 0.122 mm (Table 3). For the 3D distance the median (maximal) difference was 0.262 mm (1.040 mm). The median differences with respect to the body positions and orientations of the baseline models ranged between 0.552 and 0.739 mm and 0.96°–1.68°, respectively (Table 3). Finally, the median differences with respect to the joint positions and orientations of the baseline models ranged between 0.566 and 1.058 mm and 1.16°–1.95°, respectively (Table 3). (See also Supplementary Appendix S3 for the corresponding probability distributions.)

**TABLE 3:** Operator-dependent input parameters.

Input parameters	Median (max) X	Median (max) Y	Median (max) Z
Marker position (mm)	0.112 (0.584)	0.120 (0.717)	0.120 (1.039)
Body position (mm)	0.672 (4.71)	0.552 (3.79)	0.739 (14.74)
Body orientation (°)	1.19 (10.4)	1.68 (10.8)	0.96 (6.83)
Joint position (mm)	0.782 (4.44)	0.566 (14.32)	1.058 (12.18)
Joint orientation (°)	1.65 (16.4)	1.95 (9.97)	1.16 (7.09)

#### Kinematic Simulation Output

**FIGURE 4:**
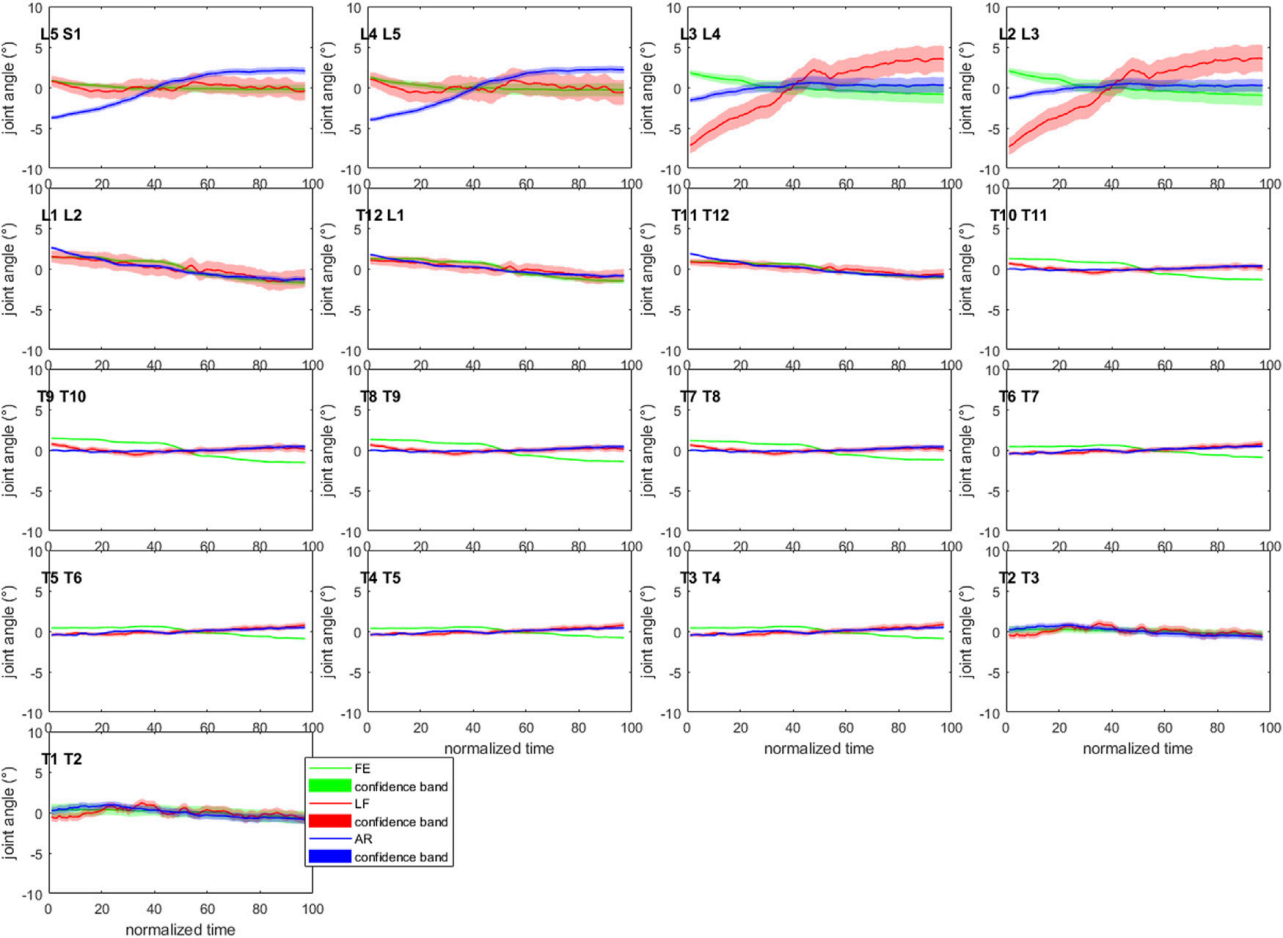
Confidence bands (5–95%) for each of the joint angles of subject 1. All curves have been normalized to their mean value over the length of the motion to allow visualization within the −10°–10° joint angle range. AR: axial rotation; LF: lateroflexion; FE: flexion-extension (Graphs for S2 and S3 are available in Supplementary Material S4.5–4.6 of Supplementary Appendix S4).

Convergence of the Monte-Carlo probabilistic simulations was reached at *n* = 954, *n* = 814 and *n* = 894 for subject S1, S2 and S3, respectively (detailed in Supplementary Appendix S4), where n is the number of iterations. For convenience, the minimal number of required iterations for convergence was rounded up to 1,000 and set equal for all subjects. Figure 4 illustrates the 90%-confidence intervals (CIs) over the duration of the motion for S1.

**FIGURE 5:**
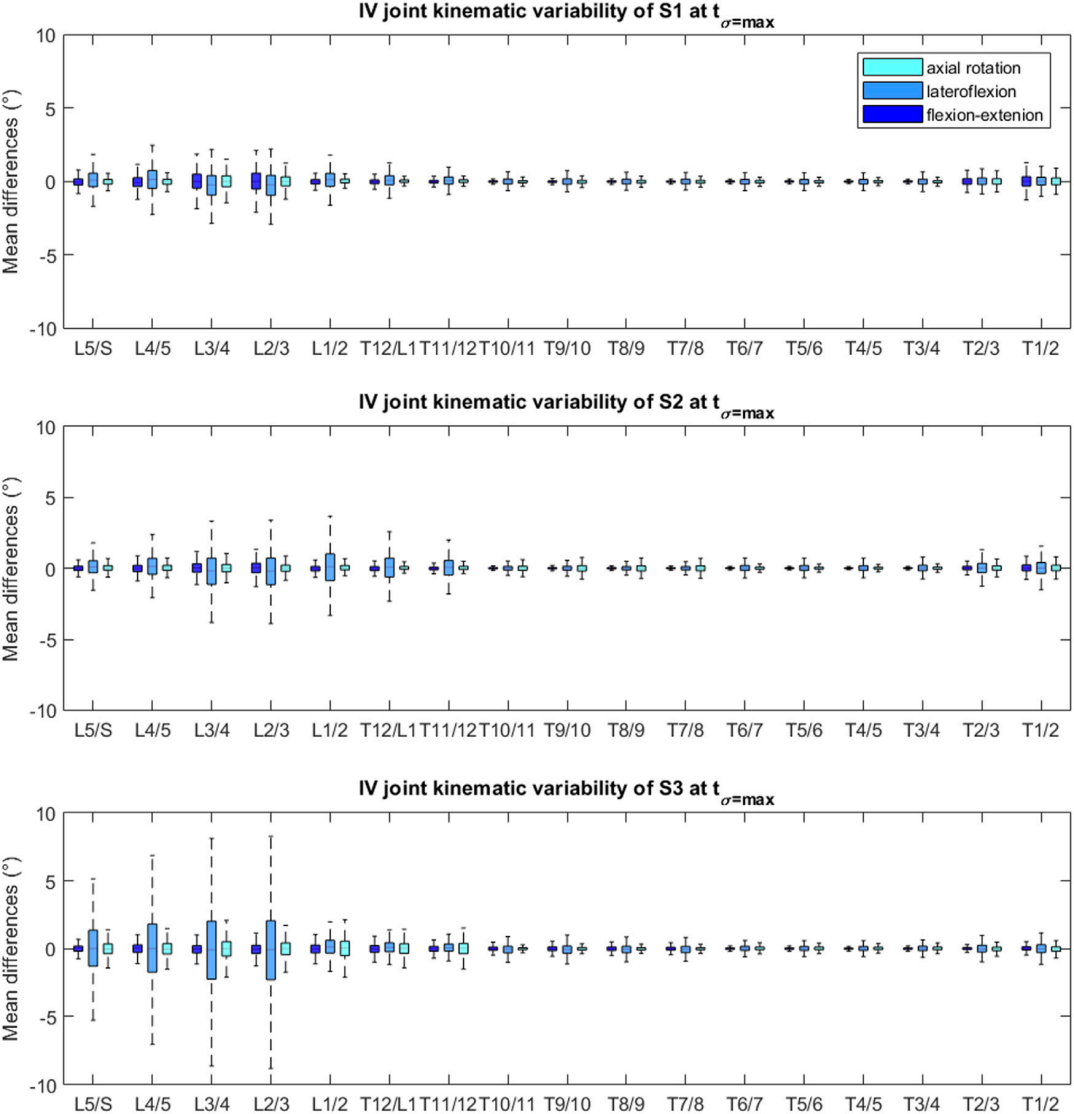
Box and whisker plot of the joint values at t_σ=max_ of each DOF, relative to the baseline model’s joint angles, for each subject. The upper and lower edges of the box are the 75th and 25th percentiles, the horizontal bar in the box is the median (50th percentile) and the upper and lower bars are maximum and minimum values.

The mean (maximum) of the 90%-CIs of the IV joint kinematics at their respective t_σ=max_ were 1.04° (3.44° at L2/L3 lateroflexion [LF]), 1.14° (4.79° at L2/L3 LF) and 1.75° (11.72° at L2/L3 LF) for S1, S2 and S3 respectively (Supplementary Figures S4.5–4.7 of Supplementary Appendix S4). The box and whisker plots show a higher variability at the lumbar and low thoracolumbar region compared to the upper thoracic region (Figure 5). Furthermore, S3 presents with larger CIs at the lumbar region than S1 and S2 (Figure 4 and Supplementary Figures S4.5–4.6 of Supplementary Appendix S4).

#### Sensitivity Factors

Calculating the sensitivity factors for all possible combinations of input (i.e., operator-dependent model parameters) and output (i.e., IV joint kinematics for every DOFs) variables, resulted in a 294 by 51 grid of correlations. Mean (maximal) sensitivity factors were 0.015 (0.15) for the marker positions, 0.015 (0.07) and 0.014 (0.06) for the body positions and orientations, respectively; and 0.022 (0.26) and 0.021 (0.47) for the joint positions and orientations, respectively.

## Discussion

This study aimed at evaluating the kinematic variability associated with both intrinsic and extrinsic sources of error (Schwartz et al., 2004), of a subject-specific spino-pelvic modeling method previously developed to quantify intervertebral joint motion in ASD subjects (Overbergh et al., 2020).

The test-retest reliability (intrinsic intra-subject and extrinsic intra-operator variability) of the kinematics within individual subjects was evaluated over a 2-week time interval. Although our method is capable of measuring spinal kinematics at the level of the IV joint, we gave priority to analyzing spino-pelvic parameters that are more commonly studied and used in clinical practice because of the lack of available literature on IV joint kinematic variability to compare to. Our results were similar to those previously reported in an ASD (Severijns et al., 2020) and healthy (Mousavi et al., 2018) population. Notably, we obtained a similar reliability for LL [ICC: 0.86 vs. 0.84 (Severijns et al., 2020) and 0.79 (Mousavi et al., 2018)] and SVA [ICC: 0.91 and 0.95 (Severijns et al., 2020)], but a lower reliability for TK [ICC: 0.12 vs. 0.95 (Severijns et al., 2020) and 0.78 (Mousavi et al., 2018)]. Although the skin marker set, pathology of the study population (ASD) and amount of subjects (5 and 8, respectively) are comparable to the study by Severijns et al. (2020), differences in the kinematic model (marker-driven subject-specific model vs. polynomial marker fit) along with the difference in motion performed by the subjects (trunk flexion [current work] vs. STS) may explain the notable difference in reliability of the TK parameter. Indeed, a maximal forward flexion is more challenging in terms of standardization compared to a STS movement. Furthermore, the thoracic region is typically more involved during maximal forward flexion compared to STS [mean ROM TK: 19.8° vs. 7.86° (Severijns et al., 2020)]. Lastly, as the modeling method is more reliant on manual operator interaction compared to Severijns et al. (2020), the modeling method may present with a potentially higher intra-operator variability, which is part of the test-retest variability.

The inter-operator kinematic agreement was assessed to investigate the effects of extrinsic inter-operator variability specifically related to the modeling method. The operator agreement in terms of spino-pelvic parameters, was excellent with ICC values ranging from 0.875 (TK) to (almost) 1 (LL, SVA, PT, T1-SPI, T9-SPI), showing a high to very high agreement amongst the three operators. Compared to Severijns et al. (2020), we report higher ICC values for LL (0.97 vs. 0.92), but slightly lower for SVA (0.964 vs. 1.00) and TK (0.875 vs. 0.91). PT, T1-SPI and T9-SPI were in almost perfect agreement. The comparable, but still slightly higher, inter-operator reliability of Severijns et al. (2020) could possibly be explained by the limited amount of operator-dependent tasks (only marker identification) in their workflow, which can be done with high accuracy (Pillet et al., 2014) compared to the additional operator-dependent tasks (i.e., CT-segmentation, marker identification, body and joint reconstruction) required to create the fully subject-specific spino-pelvic models in this work. Nevertheless, only the latter allows analysis of individual IV joint angles.

To further quantify the probabilistic effects of subject-specific spino-pelvic modeling uncertainty on intervertebral kinematics in ASD patients, we used a Monte-Carlo probabilistic simulation. The variabilities in the operator-dependent modeling parameters (i.e., the virtual markers, bodies and IV joint definition) were thereto estimated within a small group of trained operators, each creating a model of the same three ASD subjects. The operator variability in segmenting the vertebrae from CT was excluded from this study [similarly to Valente et al. (2014)] due to its previously reported high level of operator precision for lumbar vertebrae (Cook et al., 2012) and the high time cost associated with segmentation. Variability in radiograph-based virtual marker identification was small and of similar magnitude than previously reported values for a similar study (Pillet et al., 2014). Likewise, the variability in spinal alignment reconstruction (i.e., bodies component) (median position and orientation variability between 0.55 and 0.74 mm and 0.96–1.68°, respectively) was similar to the previously reported accuracy when validated with a plastinated cadaver serving as ground truth (median accuracy between 0.57 and 1.57 mm and 1.02–2.20° for vertebral positions and orientations, respectively) (Overbergh et al., 2020). The IV joint definition is based on the position and orientation of the caudal vertebral bodies and on additional landmark identification by the operator; therefore resulting in a higher median variability for the positions and orientation of the joint component (0.57–1.06 mm, 1.16–1.95°), compared to the body component. With a mean 90% CI below 2° [1.04° (S1), 1.14° (S2) and 1.75° (S3)], IV joint kinematics were found to be reliable. This is in agreement with the high reliability of the spino-pelvic parameters in our inter-operator agreement analysis. Importantly, this indicates that the modeling method as well as the resulting kinematics during forward flexion are robust towards inter-operator variability. Although, for each subject, the imposed perturbations in the model variations were sampled from the same probability distributions, different IV joint variability can be noted. Interestingly, the largest variation was consistently noted at the lumbar region (especially L2/L3) for each of the three subjects (Figure 5). This could potentially be related to a higher ROM at this region, although preliminary analyses could not confirm this due to the low number of subjects. Notably, one subject (S3) presented with more than twice as large maximal CIs (lumbar region) compared to the other two subjects. Although we need more data to confirm, this may be due to the more severe deformity of S3 (Figure 2) and associated increased sensitivity of the kinematics to modeling error. Furthermore, kinematics demonstrated very low sensitivity to marker variability (maximal sensitivity factor: 0.15). Likely, this is due to the very limited marker variability in reconstruction from x-ray (largest noted variability of 1.04 mm) compared to the traditional error associated with marker-based motion capture systems (errors of 1–5 mm, (Hicks et al., 2015)) and considerably smaller than typical skin motion artefacts [up to 10 mm for human movement, (Hicks et al., 2015)]. Very low sensitivity factors were also found for the body positions and rotations (max: 0.07 and 0.06, respectively). This can be explained by the independence of the IV joint kinematics to the alignment, provided that changes to the alignment are isolated from changes to the joint definition and virtual marker positions. Overall, the imposed variability of the IV joint positions and orientations seemed to have the biggest effect on the IV joint kinematics, with maximal sensitivity factors of 0.26 and 0.47, respectively. Consequently, this study identifies modeling steps contributing to the reliable definition of the IV joints as a primary target for limiting kinematic variability.

There are some limitations associated with this study. Firstly, the input distributions of the probabilistic simulations can vary depending on the operators and subjects, thereby affecting the simulation outputs. In this study, operator-dependent parameters were grouped as model components (i.e., the marker positions, body and joint positions and orientations) to have a sufficient amount of samples to estimate a representative probability function based on the histograms, disregarding potential variations in variability within different vertebral levels. As part of future work, a larger group of subjects with different complexities of spinal malalignments would allow a more detailed analysis of the subject-, vertebral level- and direction-dependent variability distributions. Secondly, the type of simulated motion is expected to influence the kinematic variability. Besides its clinical relevance as a task of daily living (e.g., putting on shoes), maximal forward spine flexion was used here as a worst-case scenario because of its large spinal ROM. However, one should be careful with direct extrapolation of the results presented in this study to other motions such as gait, presenting with a lower spinal range of motion, or spinal lateroflexion and axial rotation, presenting with spinal coupling, which may provide additional important insights. This uncertainty analysis focused specifically on the operator-dependent components of the modeling method, thereby ignoring additional variability, for example originating from inter-rater variability in skin marker placement. Lastly, our uncertainty analysis was limited to IV joint kinematics as outcome. However additional analyses should be done to assess the uncertainty propagation in possible subsequent simulation steps such as joint reaction forces or muscle activation (Myers et al., 2015; Burkhart et al., 2020).

Our systematic inter-operator approaches identified a limited impact of operator-induced variability on kinematic simulations of spine flexion in an ASD population. This excellent inter-operator agreement, compared to the lower test-retest reliability for the same motion, however, importantly indicates that the dominant portion of overall test-retest variability is only limitedly originating from aspects of the modeling (extrinsic), but rather from intra-subject differences (intrinsic) in motor task execution. Improved standardization of the maximal forward trunk flexion (e.g., pelvic fixation and/or targets) together with multiple acquisitions averaged per session, may thus improve the test-retest reliability.

In conclusion, although the current modeling method is dependent on manual inputs of the operators, causing additional variability in the simulation output, its isolated effect on the kinematics was very limited, indicating the modeling method to be highly reliable for kinematic analysis of spinal motion. In the future, this kinematic variability could likely be even further reduced by eliminating variability in operator-dependent model components through increased automation of the model creation procedures. Furthermore, this would also decrease the currently high time cost of subject-specific modeling (Aubert et al., 2019; Galbusera et al., 2020). Based on this study’s results, the primary focus should hereby be on the intervertebral joint definition.

## Data Availability

The raw data supporting the conclusion of this article will be made available by the authors, without undue reservation.
