# Supplementary material for: Subject-Specific Spino-Pelvic Models Reliably Measure Spinal Kinematics During Seated Forward Bending in Adult Spinal Deformity
Source: Front Bioeng Biotechnol. 2021 Sep 1;9:720060. doi: 10.3389/fbioe.2021.720060 (PMC8440831; doi:10.3389/fbioe.2021.720060)
Supplement: Supplementary file 1 [file DataSheet1.pdf]

Supplementary Material

1 Appendix 1: Spino-pelvic parameterization of joint kinematics

1.1 Calculating dynamic spino-pelvic parameters

Joint kinematics (i.e. relative motion at the joint between two interconnected bodies) were converted to body kinematics (i.e. absolute motion of a body expressed in a fixed ground reference frame) using the API of OpenSim 3.3 (Stanford University, USA). Thereafter, anatomical landmarks on the bodies previously indicated during the mesh-based IV joint definition when creating the model are used (Overbergh et al., 2020). As the location of a landmark on a body is fixed during the motion, its transformation matrix was appended to the body kinematics to obtain the 3D trajectory of each anatomical landmark throughout the trunk flexion motion. Thereafter, six common spino-pelvic parameters in the sagittal plane, i.e. lumbar lordosis (LL), thoracic kyphosis (TK), sagittal vertical axis* (SVA, Figure 1.1), pelvic tilt (PT) and T1 and T9 spino-pelvic inclination (T1-SPI, T9-SPI) were calculated for every time frame (detailed in Table 1.1). The acetabular cavities of pelvis were used to define the sagittal and coronal plane in which the spino-pelvic parameters are expressed (Figure 1.1.B).

Supplementary Table 1.1. Description of the model-based spino-pelvic parameterization.

**Spino-pelvic parameter (unit)**	**Model-based calculation**
Lumbar lordosis (LL, °)	Calculated from the sagittal plane projection of the four-points angle defined by the line connecting the anterior and posterior landmarks of the T12 superior endplate, and the line connecting the anterior and posterior landmarks of the sacral endplate.
Thoracic kyphosis (TK, °)	Calculated from the sagittal plane projection of the four-points angle defined by the line connecting the anterior and posterior landmarks of the T1 superior endplate, and the line connecting the anterior and posterior landmarks of the inferior T12 endplate.
Sagittal vertical axis (SVA, cm)	The distance between the posterior sacral endplate and the plumb line of the T1 vertebral body, measured in the transverse plane and perpendicular to the line connecting the left and right acetabula (Figure 1.1).<br><br>**Because our model is limited to the bodies from pelvis up to T1, the T1 vertebral body was used rather than the C7 vertebral body for the calculation of the SVA.*
Pelvic tilt (PT, °)	The projection on the sagittal plane of the three-points angle defined by the vertical line on the mid-point of the line connecting the left and right acetabula, and the line then going to the midpoint of the anterior and posterior sacral endpoint.
T1-Spino-pelvic inclination (T1-SPI, °)	The projection on the sagittal plane of the three-points angle defined by the line starting on the mid-point of the line connecting the left and right acetabula, to the T1 vertebral body center, with a vertical line from the T1 vertebral body center.
T9-Spino-pelvic inclination (T9-SPI, °)	The projection on the sagittal plane of the three-points angle defined by the line starting on the mid-point of the line connecting the left and right acetabula, to the T9 vertebral body center, with a vertical line from the T9 vertebral body center.

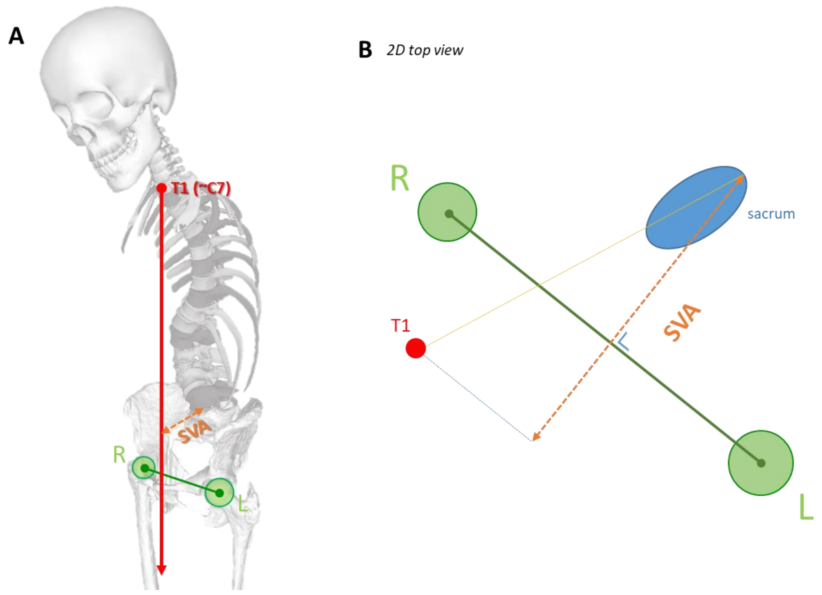

Supplementary Figure 1.1. (A) A visual representation on the model and (B) schematic of the sagittal vertical axis (SVA) as described in Table 1.1.

1.2 Calculating range of motion

To determine the range of motion (ROM) of these spino-pelvic parameters over the duration of the trunk flexion motion, the absolute difference between the values at the start (mean of the first three frames) and end (mean of the last three frames) of the motion were used. Due to noise on the kinematic simulation output, a different value would have been obtained if ROM would have been calculated based on the minimal and maximal values, who's bounds would not necessarily encloses the complete motion.

2 Appendix 2: Training the alignment reconstruction

The operators were allowed to familiarize with the modeling software (Overbergh et al., 2020), guided by a written manual, using the images of the control subject, until they felt confident working with the software. The operator training was concluded with the alignment reconstruction of a plastinated cadaver as in (Overbergh et al., 2020). The plastinated cadaver was rigidly fixated, preventing the spinal alignment to change between upright biplanar images, used for alignment reconstruction, and the supine CT, used as ground truth for evaluating the alignment reconstruction accuracy. All operators performed this test, however, the results of the reconstruction accuracy of operator 1 (O1) were previously published (Overbergh et al., 2020). The same randomized start model was provided to the operators (Figure 2.1.A). After reconstruction, the results were compared to the CT-segmented ground truth (Figure 2.1.B, Table 2.1) for each operator.

The error values for the newly trained operators (O2 and O3) were consistently higher compared to the error value of O1, who developed the method.

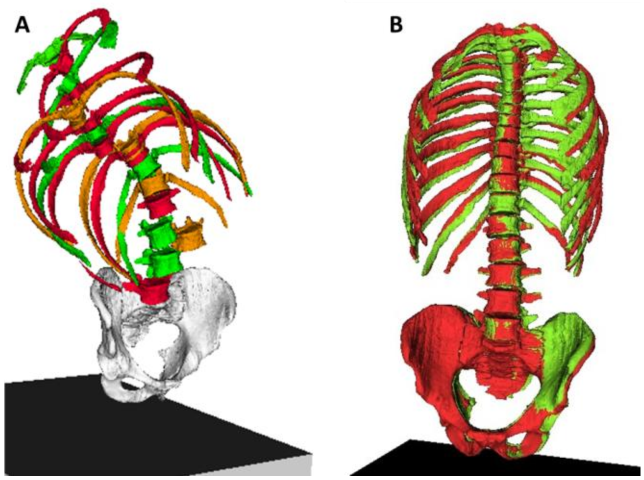

Supplementary Figure 2.1. Illustration of (A) the randomized initial model of the plastinated cadaver and (B) the end result of the model reconstructed by operator 2 (O2, red) superimposed on the CT-segmented ground truth (green).

Supplementary Table 2.1. The root mean square (RMS) values of the absolute (abs.) reconstruction error for each of the three operators (O1-3) (M-L = mediolateral, I-S = inferosuperior, A- P = anteroposterior).

	RMS O1 (Overbergh et al., 2020)	RMS O2	RMS O3
Abs. sagittal orientation (°)	1.19	5.00	4.72
Abs. transverse orientation (°)	1.91	3.87	3.81
Abs. coronal orientation (°)	0.76	2.21	4.47
3D distance (mm)	1.26	6.03	4.33
Abs. M-L position (mm)	0.51	2.82	3.21
Abs. I-S position (mm)	0.89	4.42	1.88
Abs. A-P position (mm)	0.87	2.97	2.23

3 Appendix 3: Distributions of the variability on the operator-dependent model parameters

The Shapiro-Wilk test was used to test the normality of the parameters (position and orientation) of the model components (markers, bodies, joints), which indicated non-normal distributions of the variability in virtual marker position (Table 3.1) at the 0.05 significance level (SPSS 26, IBM). Also for the body positions and orientations (Table 3.2) and the joint positions and orientations (Table 3.3), the parameters did not all have a normal distribution. Thereafter, kernel functions were consistently used to estimate all distribution functions from the respective error histograms (Figures 3.1-3.3) (Distribution Fitter, MATLAB, The Mathworks Inc., MA).

3.1 Virtual markers

Supplementary Table 3.1. Shapiro-Wilk test for normality on the parameters defining the markers (X: medio-lateral, Y: infero-superior, Z: antero-posterior)

	Statistic	Significance
X position	0.983	0.005
Y position	0.976	0.000
Z position	0.961	0.000

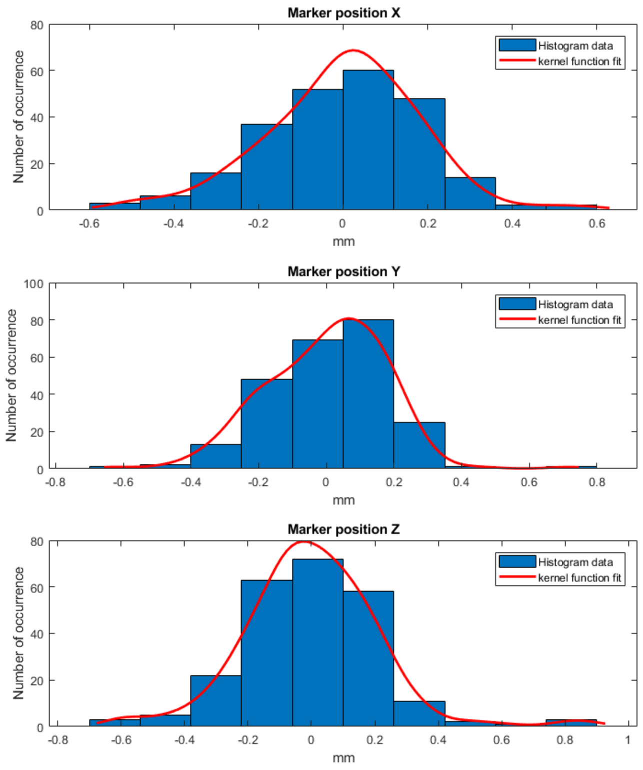

Supplementary Figure 3.1. The histograms of the marker position differences relative to the baseline models, for each direction, with fitted kernel functions. (X: medio-lateral, Y: infero-superior, Z: antero-posterior)

3.2 Bodies

Supplementary Table 3.2. Shapiro-Wilk test for normality of the parameters defining the bodies (X: medio-lateral, Y: infero-superior, Z: antero-posterior)

	Statistic	Significance
position X	0.903	0.001
position Y	0.962	0.104
position Z	0.965	0.139
orientation X	0.955	0.054
orientation Y	0.967	0.161
orientation Z	0.981	0.570

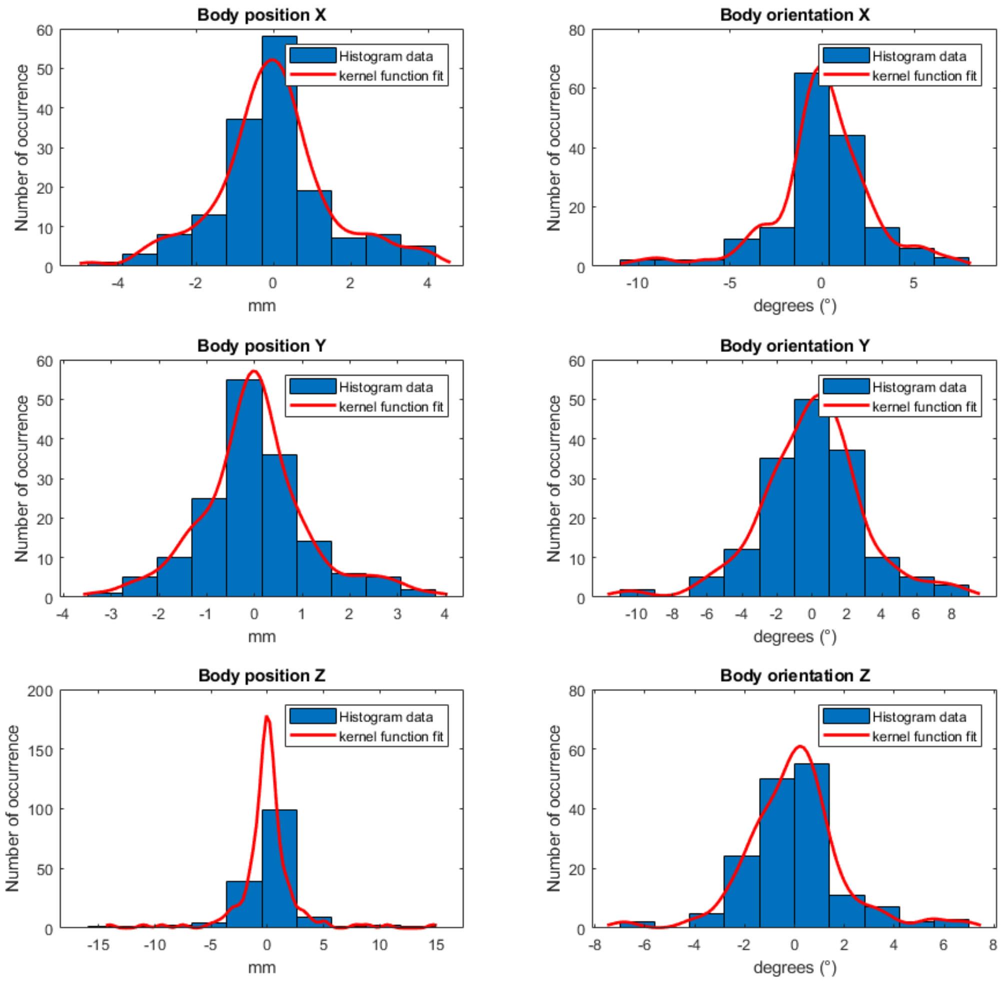

Supplementary Figure 3.2. The histograms of the body position and orientation differences relative to the baseline models, for each direction, with fitted kernel functions. (X: medio-lateral, Y: infero-superior, Z: antero-posterior)

3.3 Joints

Supplementary Table 3.3. Shapiro-Wilk test for normality on the parameters defining the joints (X: medio-lateral, Y: infero-superior, Z: antero-posterior)

	Statistic	Significance
position X	0.977	0.010
position Y	0.736	0.000
position Z	0.834	0.000
orientation X	0.934	0.000
orientation Y	0.983	0.048
orientation Z	0.970	0.002

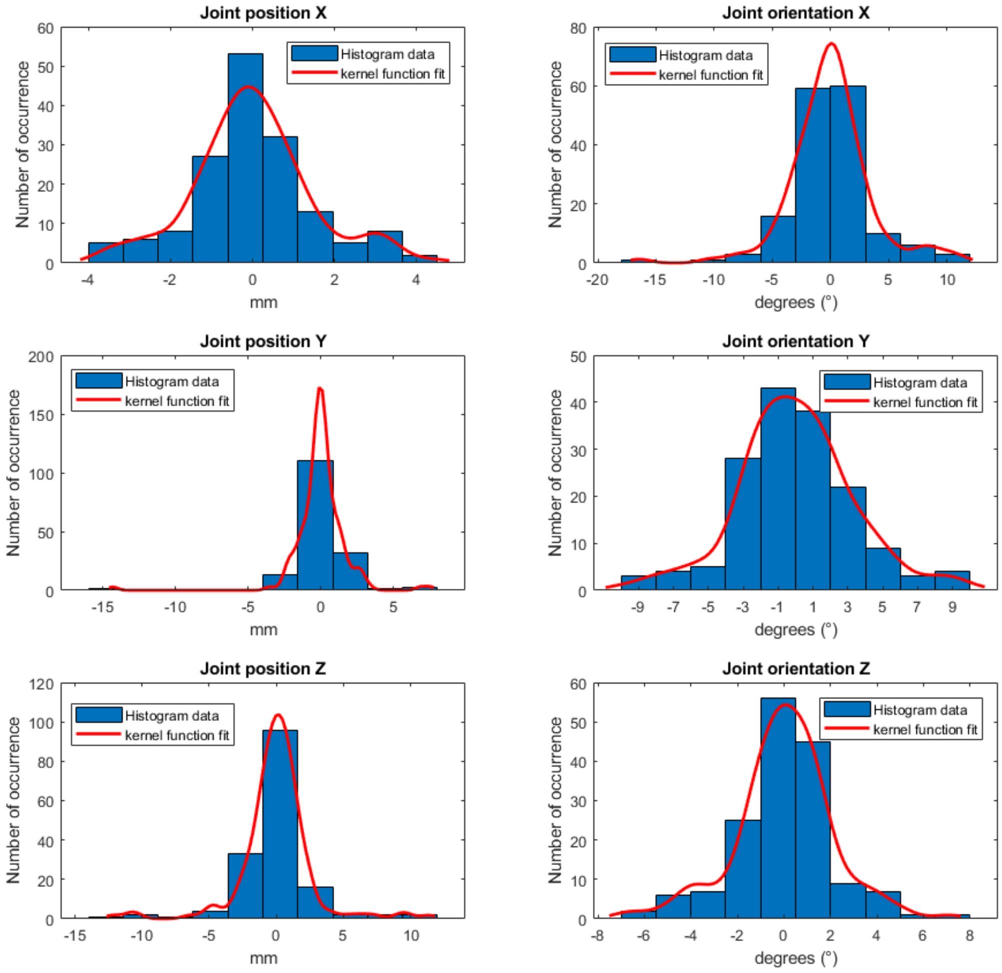

Supplementary Figure 3.3. The histograms of the joint position and orientation differences relative to the baseline models, for each direction, with fitted kernel functions. (X: medio-lateral, Y: infero-superior, Z: antero-posterior)

4 Appendix 4: The Monte-Carlo probabilistic simulation

4.1 Stop criteria and convergence

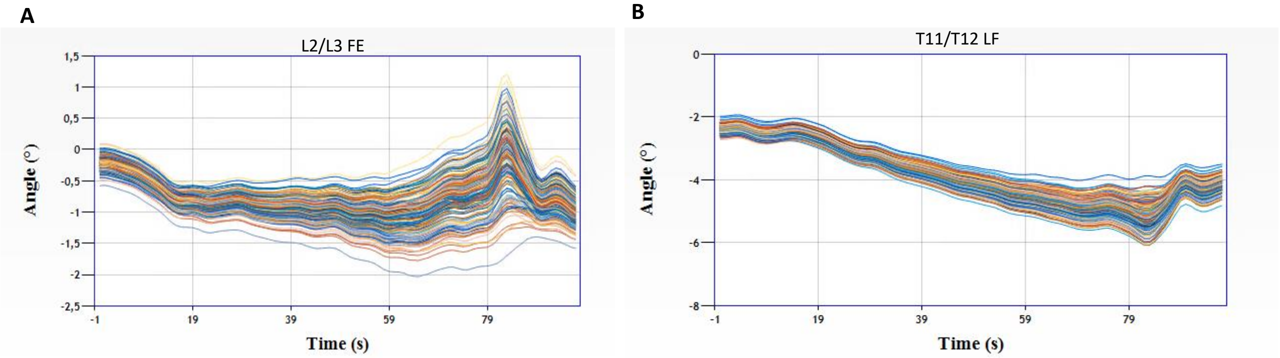

Supplementary Figure 4.1. Illustration of 1000 intervertebral (IV) joint kinematics curves for (A) the L2/L3 IV joint in the flexion-extension (FE) degree of freedom (DOF) and (B) for the T11/T12 IV joint in the lateroflexion (LF) DOF.

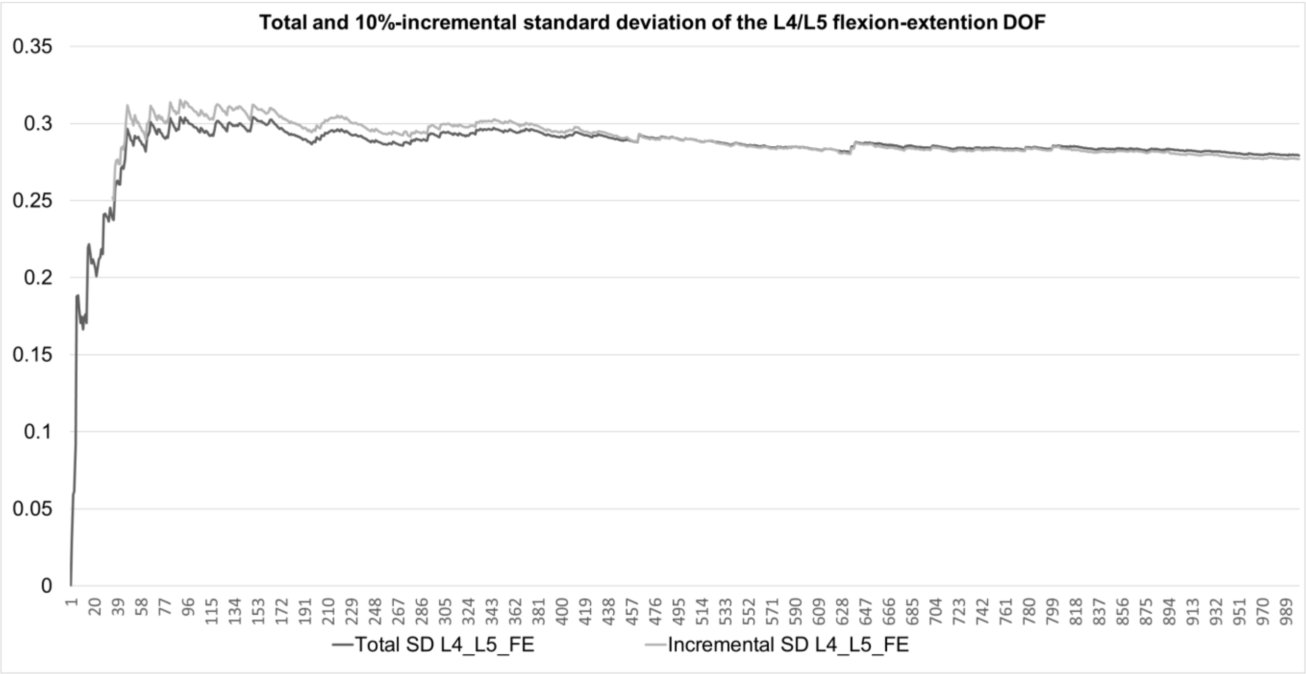

Supplementary Figure 4.2. The total standard deviation (SD, blue) at each iteration over all samples and the SD computed over the last 10% of samples (orange), up to that iteration index (x-axis). As the amount of iterations increases, the difference between the total SD and the SD over the last 10% of samples becomes smaller (see also Figure 4.3 for a details on the instance of convergence).

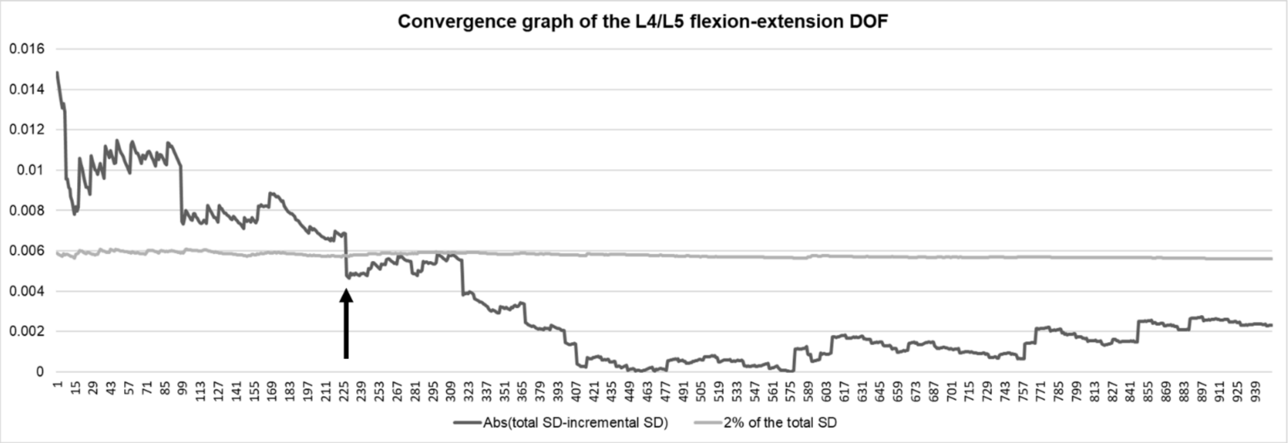

Supplementary Figure 4.3. The absolute difference between the total standard deviation (SD) (i.e. over all iterations up to that iteration index) and the SD calculated only over the last 10% of iterations (blue). 2% of the total SD calculated (orange). At about 220 iterations the L4/L5 flexion-extension joint angle converges according to the SD stop-criterion.

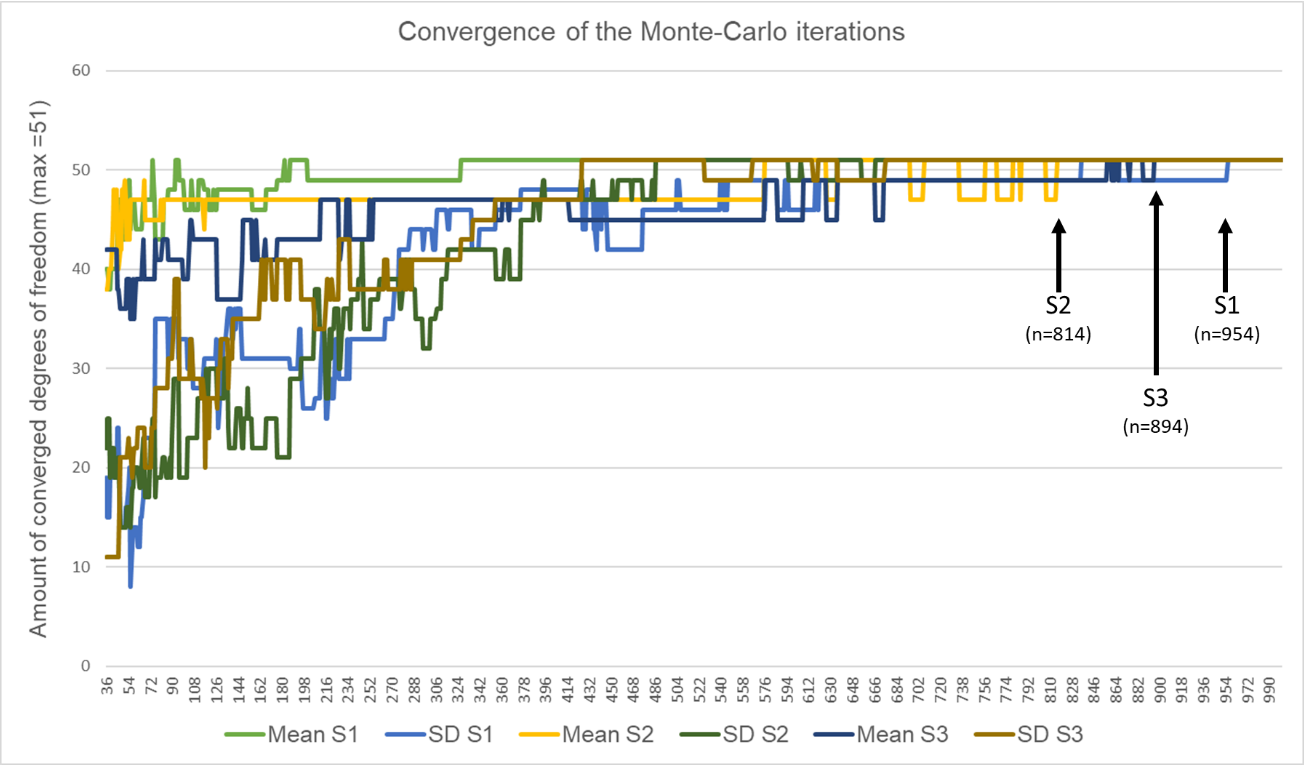

Supplementary Figure 4.4. Convergence for Subject 1 (S1), Subject 2 (S2) and Subject 3 (S3) according to the stop criteria on the mean and standard deviation (SD). Convergence is reached at the iteration index (n) (x-axis) where both the stop criteria (i.e. mean and SD) have converged for all of the 51 degrees of freedom (y-axis) and was indicated on the graph for each subject.

Supplementary Table 4.5. Iteration indexes at which convergence occurs according to the mean and standard deviation stop criteria. The maximal index (bold) of both criteria determines the convergence index for each subject (S1-S3).

Subject	Iteration index of convergence according to the *mean* criterion	Iteration index of convergence according to the *standard deviation* criterion
S1	325	**954**
S2	**814**	665
S3	**894**	673

4.2 Intervertebral joint kinematics

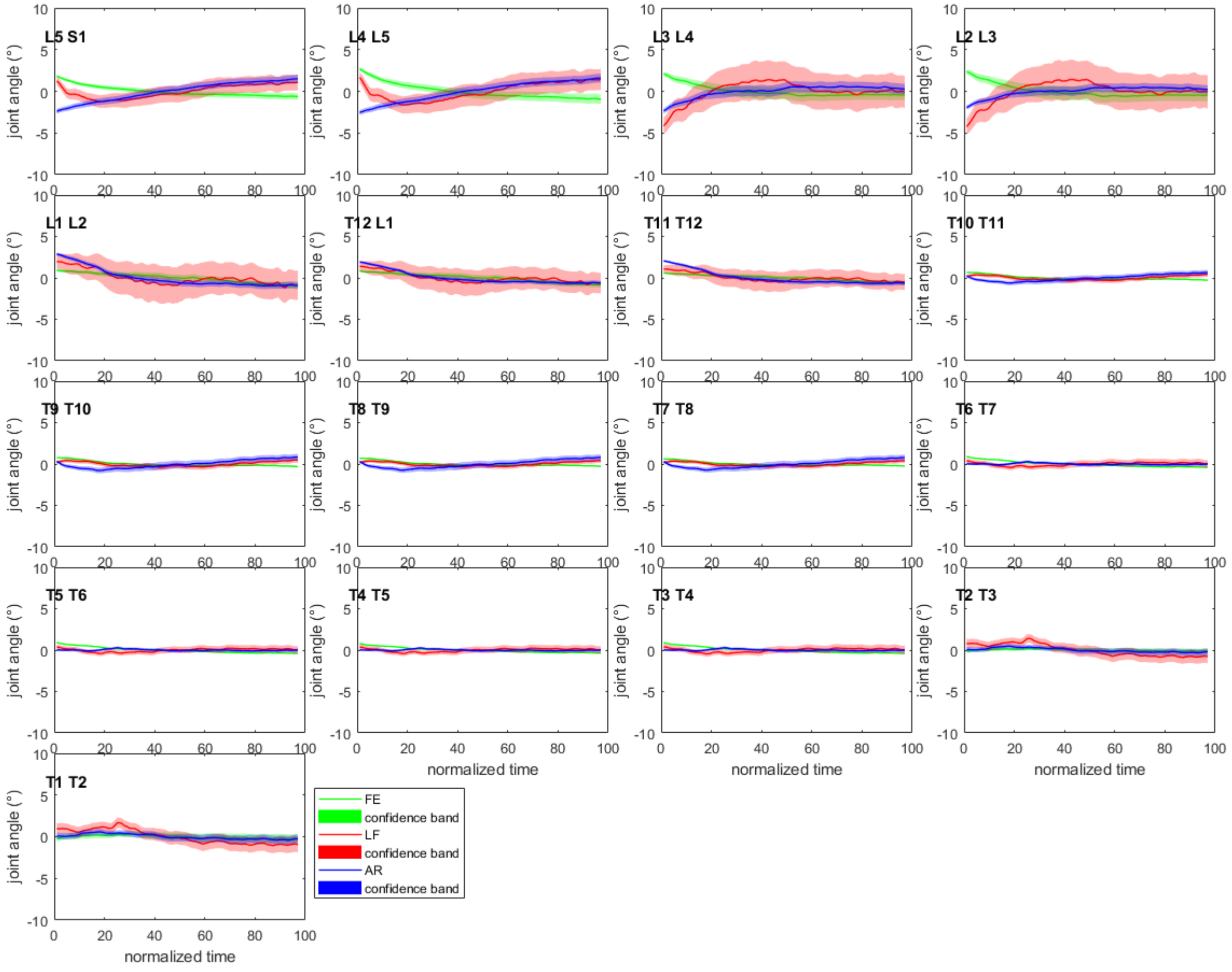

Supplementary Figure 4.5. Confidence bands (5-95%) for each of the joint angles for subject 2 (S2). All curves have been normalized to their mean value over the length of the motion to allow visualization within the -10° to 10° joint angle range. AR: axial rotation; LF: lateroflexion; FE: flexion-extension.

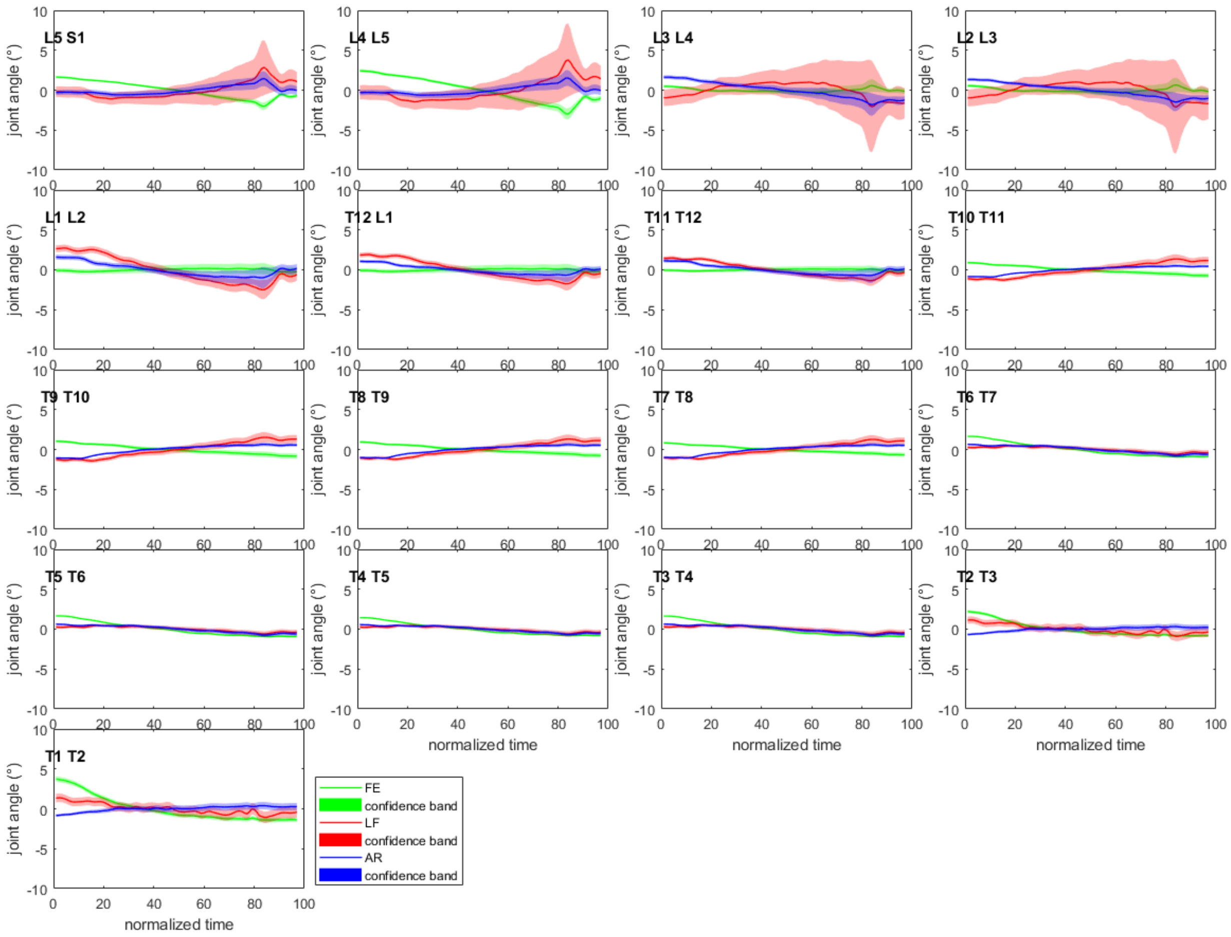

Supplementary Figure 4.6. Confidence bands (5-95%) for each of the joint angles of subject 3 (S3). All curves have been normalized to their mean value over the length of the motion to allow visualization within the -10° to 10° joint angle range. AR: axial rotation; LF: lateroflexion; FE: flexion-extension.

Supplementary Table 4.6. Time instance (ranging between 1 and 100) of maximal variance ($t_{\sigma=max}$) for the individual joint angles (FE: flexion-extension, LF: lateroflexion, AR: axial rotation) and subjects (S1-S3).

Degrees of freedom	S1	S2	S3
L5_S1_FE	97	100	87
L5_S1_LF	93	59	86
L5_S1_AR	99	100	87
L4_L5_FE	97	100	87
L4_L5_LF	93	59	86
L4_L5_AR	99	100	87
L3_L4_FE	99	51	86
L3_L4_LF	93	40	86
L3_L4_AR	99	45	86
L2_L3_FE	99	51	86
L2_L3_LF	93	40	86
L2_L3_AR	99	45	86
L1_L2_FE	91	53	86
L1_L2_LF	92	44	87
L1_L2_AR	99	84	86
T12_L1_FE	91	53	86
T12_L1_LF	92	44	87
T12_L1_AR	99	84	86
T11_T12_FE	91	53	86
T11_T12_LF	92	44	87
T11_T12_AR	99	84	86
T10_T11_FE	100	19	87
T10_T11_LF	92	44	86
T10_T11_AR	100	44	86
T9_T10_FE	100	19	87
T9_T10_LF	92	44	86
T9_T10_AR	100	44	86
T8_T9_FE	100	19	87
T8_T9_LF	92	44	86
T8_T9_AR	100	44	86
T7_T8_FE	100	19	87
T7_T8_LF	92	44	86
T7_T8_AR	100	44	86
T6_T7_FE	100	96	87
T6_T7_LF	98	97	87
T6_T7_AR	90	83	87
T5_T6_FE	100	96	87
T5_T6_LF	98	97	87
T5_T6_AR	90	83	87
T4_T5_FE	100	96	87
T4_T5_LF	98	97	87
T4_T5_AR	90	83	87
T3_T4_FE	100	96	87
T3_T4_LF	98	97	87
T3_T4_AR	90	83	87
T2_T3_FE	100	90	2
T2_T3_LF	96	98	87
T2_T3_AR	100	97	87
T1_T2_FE	100	90	2
T1_T2_LF	96	98	87
T1_T2_AR	100	97	87
